# MicroRNA 132-3p Is Upregulated in Laron Syndrome Patients and Controls Longevity Gene Expression

**DOI:** 10.3390/ijms222111861

**Published:** 2021-11-01

**Authors:** Danielle Yaron-Saminsky, Karthik Nagaraj, Rive Sarfstein, Zvi Laron, Metsada Pasmanik-Chor, Haim Werner

**Affiliations:** 1 Department of Human Molecular Genetics and Biochemistry, Sackler School of Medicine, Tel Aviv University, Tel Aviv 69978, Israel; danielle.yaron@gmail.com (D.Y.-S.); mailkartz@gmail.com (K.N.); rives@tauex.tau.ac.il (R.S.); 2 Schneider Children’s Medical Center, Endocrinology and Diabetes Research Unit, 49292 Petah Tikva, Israel; laronz@clalit.org.il; 3 Bioinformatics Unit, The George S. Wise Faculty of Life Sciences, Tel Aviv University, 69978 Tel Aviv, Israel; metsada@tauex.tau.ac.il

**Keywords:** microRNA-132-3p, insulin-like growth factor-1 (IGF1), Laron syndrome, congenital IGF1 deficiency, longevity, SIRT1

## Abstract

The growth hormone (GH)–insulin-like growth factor-1 (IGF1) endocrine axis is a central player in normal growth and metabolism as well as in a number of pathologies, including cancer. The GH–IGF1 hormonal system, in addition, has emerged as a major determinant of lifespan and healthspan. Laron syndrome (LS), the best characterized entity under the spectrum of the congenital IGF1 deficiencies, results from mutation of the GH receptor (GHR) gene, leading to dwarfism, obesity and other defects. Consistent with the key role of IGF1 in cellular proliferation, epidemiological studies have shown that LS patients are protected from cancer development. While reduced expression of components of the GH-IGF1 axis is associated with enhanced longevity in animal models, it is still unknown whether LS is associated with an increased lifespan. MicroRNAs (miRs) are endogenous short non-coding RNAs that regulate the expression of complementary mRNAs. While a number of miRs involved in the regulation of IGF components have been identified, no previous studies have investigated the differential expression of miRs in congenital IGF1 deficiencies. The present study was aimed at identifying miRs that are differentially expressed in LS and that might account for the phenotypic features of LS patients, including longevity. Our genomic analyses provide evidence that miR-132-3p was highly expressed in LS. In addition, we identified SIRT1, a member of the sirtuin family of histone deacetylases, as a target for negative regulation by miR-132-3p. The data was consistent with the notion that low concentrations of IGF1 in LS lead to elevated miR-132-3p levels, with ensuing reduction in SIRT1 gene expression. The impact of the IGF1-miR-132-3p-SIRT1 loop on aging merits further investigation.

## 1. Introduction

Insulin-like growth factor-1 (IGF1) plays important roles in the regulation of metabolism, nutrition and growth [1,2]. Endocrine and tissue IGF1 levels are tightly controlled throughout development, with peak levels typically reached at puberty and reduced during adulthood and aging [3,4,5]. Circulating IGF1 levels are dependent on liver production, which is governed by pituitary-derived growth hormone (GH) [6,7,8,9]. Elevated systemic levels of IGF1 correlate with an increased risk of developing a number of malignancies, including breast and prostate cancer [10,11,12,13]. Likewise, overexpression of the cell-surface IGF1 receptor (IGF1R) has been consistently linked to malignant transformation [14,15]. Enhanced expression of components of the IGF system in cancer cells reflects the potent mitogenic, anti-apoptotic and pro-survival activities of this growth factor axis [16,17,18,19].

Laron syndrome (LS), or primary GH insensitivity, is a recessively transmitted genetic form of dwarfism caused by the deletion or mutation of the GH receptor (*GHR*) gene, leading to congenital IGF1 deficiency [20,21,22]. The typical features of LS are short stature, typical face, obesity, high basal serum GH and low IGF1, unresponsive to the administration of exogenous GH [23,24]. The recognition that an inherited defect of the *GHR* gene, including exon deletions and point mutations, is the etiological factor behind LS was reported in 1989 [25,26].

Epidemiological studies conducted by one of us evaluated whether LS patients have a reduced cancer prevalence [27,28]. The rationale for this quest was the fact that elevated IGF1, as alluded to above, is regarded as a risk factor for cancer. On the other hand, the impact of low IGF1 on cancer prevalence has not yet been explored in a systematic fashion. Indeed, analyses revealed that the rate of cancer incidence in LS patients is extremely low. Protection from cancer was also reported in an Ecuadorian cohort of LS patients [29]. Reduced activities of the GHR, IGF1R and downstream signaling mediators (e.g., AKT, mTOR, FOXO) have been consistently correlated with increased lifespan [30,31,32]. Thus, studies on GHR-KO (‘*Laron*’) mice generated evidence that these animals are exceptionally long-lived compared to their wild type littermates [33,34,35]. It is unknown whether LS is associated with noticeable longevity [36,37].

Genome-wide profiling aimed at identifying genes and signaling pathways that are differentially represented in LS and that may account for some of the distinctive features of the disease, including cancer protection, were recently conducted on patients, relatives and healthy controls of the same ethnic groups [38]. Analyses identified a series of highly expressed metabolic genes, including thioredoxin-interacting protein (TXNIP), zyg-11 homolog A (ZYG11A), etc. The roles of some of these novel genes in the context of LS pathogenesis and, in particular, the putative cancer-protective tasks of these gene products were recently described [39,40]. In addition, a number of genes usually involved in growth promotion were shown to be under-represented in LS [41,42]. Of notice, these genes have not been previously linked to the IGF1 signaling axis [43].

MicroRNAs (miRs) are endogenous short non-coding RNAs that regulate the expression of complementary mRNAs [44,45,46]. MiRs are able to pair to specific protein-coding mRNAs, leading to post-transcriptional repression of target genes. miRs exert multiple roles in several biological processes, including developmental timing, cell death and proliferation, hematopoiesis and patterning of the nervous system [47,48]. At present, over 2000 miRs have been discovered in humans and it is believed that, collectively, they regulate about one third of the genes in the entire genome.

In the specific context of the IGF1 signaling axis, several miRs involved in the regulation of IGF components have been identified [49,50,51,52]. Pathologic dysregulation of some of these miRs was associated with IGF1R overexpression, growth and metabolic dysfunction, and malignant transformation [53,54]. MicroRNA 132-3p (hsa-miR-132-3p, or in short, miR-132-3p or miR-132) has been the topic of extensive investigation in recent years [55]. MiR-132-3p is regarded as a key miR and was shown to interact with a number of target genes, affecting various biological functions (e.g., inflammation, angiogenesis, neuronal differentiation, etc.) [56]. Of interest, high expression of miR-132-3p in conjunction with low levels of SIRT1 was reported in lymphoblastoids of Alzheimer’s disease patients [57].

The present study was aimed at identifying miRs that are differentially expressed in LS and that might account for the phenotypic features of LS patients, including cancer protection and, possibly, longevity. No previous studies have explored the putative over- or under-representation of specific miRs in congenital IGF1 deficiencies. In addition, and in view of the enhanced expression of miR-132-3p in LS-derived cells, we examined the impact of miR-132-3p expression on a number of aging markers in vitro. Our data identified a series of important regulatory activities associated with miR-132-3p. The biological and clinical implications of these novel findings merit further investigation.

## 2. Results

### 2.1. Genome-Wide Analysis of Differentially Expressed miRs in LS

**Figure 1:**
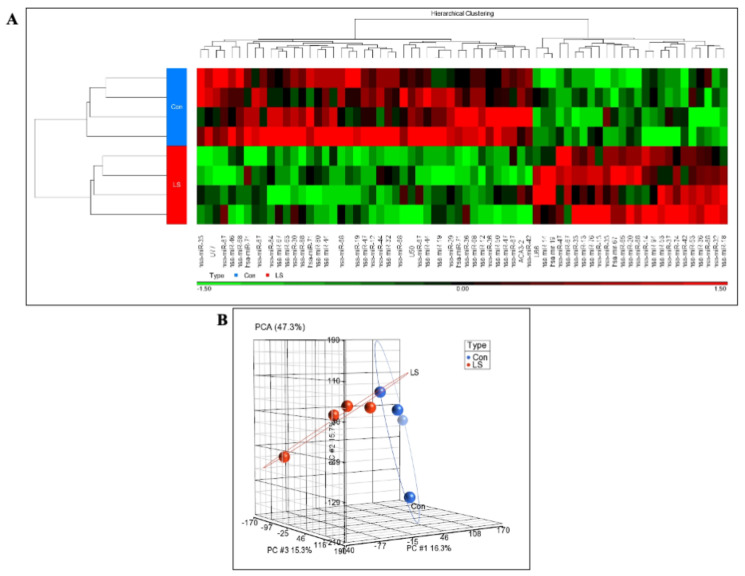
Genome-wide microRNA profiling of LS patients. (**A**) Hierarchical cluster analysis (Pearson’s Dissimilarity, Ward’s method) performed for 68 differentially-expressed miRs (*p* < 0.05 and fold-change difference = 1.5), with four LS and four control samples. (**B**) Principal component analysis (PCA) display of four LS (red dots) and four control (blue dots) samples used in the experiment. PCA demonstrated a good discrimination between groups.

Recently performed global profiling assays identified the entire collection of genes and signaling pathways that were differentially represented in LS and that may explain, at least partly, phenotypic features associated with this condition, including cancer protection. To identify non-coding miRs that were either over- or under-expressed in LS, we adopted a similar global approach using GeneChip^®^ miRNA arrays that include the entire repertoire of small non-coding RNA transcripts. Total RNA was obtained from individual EBV-immortalized lymphoblastoid cell lines derived from four female LS patients and four controls of the same ethnic origin (Iraq, Yemen, Iran) and age range (LS, 44.25 ± 6.08 years; controls, 51.75 ± 11.3 years; mean ± SD; *p*-value = 0.29). One-way ANOVA was performed using Partek® Genomics Suite software to create a list of differentially expressed miRs. A cluster analysis of differentially expressed miRs is depicted in Figure 1A. Sixty-eight human miRs were differentially expressed in LS versus controls (fold-change difference = 1.5 and *p* < 0.05). To summarize replicate quality and differences between conditions, principal component analysis (PCA) was conducted. As shown in Figure 1B, PCA revealed a good discrimination between experimental groups.

MiR data was combined with the 130 differentially-expressed genes from previous genome-wide mRNA analysis (same cutoff) using Partek GS along with TargetScan database for target prediction [38]. Only miR-gene targets that were negatively correlated were selected. Data is summarized in Table 1. Data identified seven miRs (hsa-miR-132-3p, hsa-miR-362-5p, hsa-miR-421, hsa-miR-200c-3p, hsa-miR-532-5p, hsa-miR-199a-3p, hsa-miR-30b-5p) that targeted 13 genes experimentally obtained by gene expression (*AKT3, NMT2, IL6ST, MXI1, NPNT, THRA, SLC7A11, DCBLD2, CCNE2, FUCA1, FZD3, RGL1, TRERF1*). A few of the miRs targeted several genes and some genes were regulated by more than one miR.

### 2.2. Identification of miR-132-3p as a Differentially Expressed miR in LS

**Figure 2:**
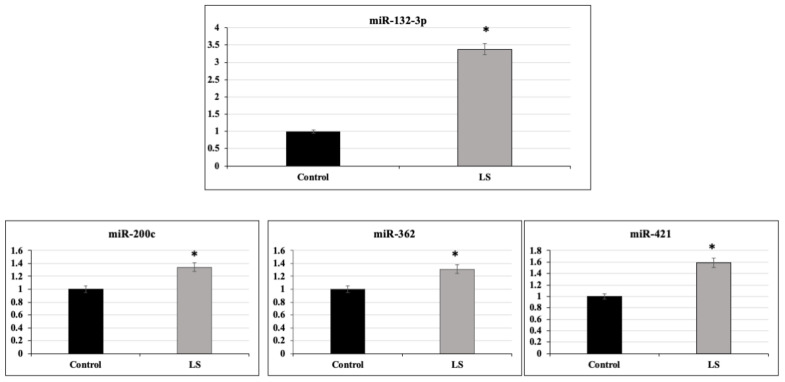
Validation analysis of selected miRs. Selected miRs that display fold-changes larger than 1.5 between LS patients and controls in microRNA arrays were selected for further validation by qRT-PCR. The selected miRs include: miR-132-3p, miR-421-3p, miR-200c-3p and miR-362-5p. Total RNA was prepared from EBV-immortalized lymphoblastoid cells derived from patients and controls analyzed in microRNA arrays; miRs levels were measured using appropriate primers, as described in the Section 4. * *p* < 0.01 versus respective control cells.

To validate the differences in miR expression between LS patients and controls identified in miRNA arrays, qRT-PCR was performed on miR-132-3p, miR-421-3p, miR-200c-3p and miR-362-5p. The rationale for this selection was the fact that these specific miRs exhibited high and significant differences between LS and control cells in miRNA arrays, as revealed by their *p*-value (Table 1). Results of qRT-PCR assays indicate that all four miRs were significantly upregulated in LS compared to control cells (Figure 2). The differences between LS and control cells in miR-421-3p, miR-200c-3p and miR-362-5p, while statistically significant, were much smaller than the differences seen with miR-132-3p. Therefore, this last miR was selected for further analyses (Figure 2).

### 2.3. Analysis of miR-132-3p Target mRNAs Expression in LS Cells

**Figure 3:**
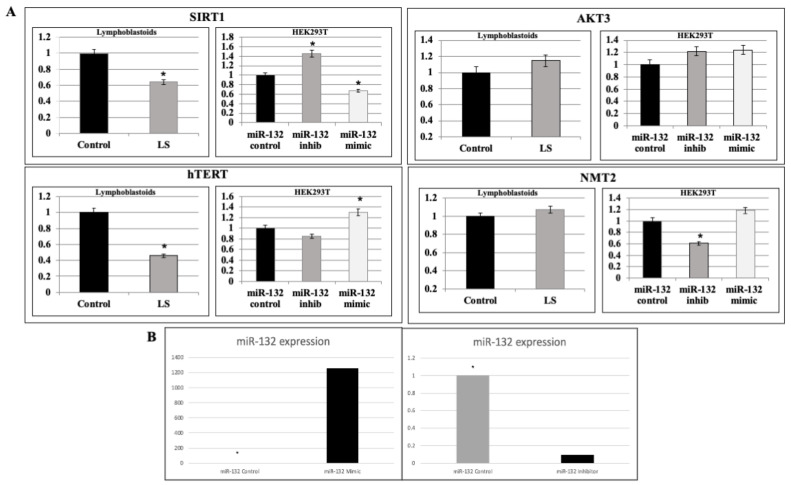
Expression of miR-132-3p target genes. (**A**) Total RNA was prepared from four individual LS patient-derived lymphoblastoid cultures and four control cultures. Levels of *SIRT1, hTERT, AKT3* and *NMT2* mRNAs were determined by qRT-PCR. For each of the four genes, the left panel represents measurements obtained in EBV-immortalized lymphoblastoid cell lines (solid bars, control cultures; gray bars, LS patients) and the right panel denotes measurents in *mir*Vana™ miRNA-transfected HEK293T cells. A value of 1 was given to the mRNA level in control cultures. HEK293T cells were transfected with *mir*Vana™ miRNA-132-3p inhibitor (gray bars) or mimics (open bars) for 24 h, after which, specific mRNA expression was assessed by qRT-PCR. A value of 1 was given to the specific mRNA levels in control-transfected cells (solid bars). * *p* < 0.01 versus respective control cells. (**B**) Expression of miR-132-3p in miR-132-3p inhibitor or mimic-transfected HEK293T cells. A value of 1 was given to the miR-132-3p levels in control-transfected cells.

Next, we examined the endogenous expression of a number of genes shown by bioinformatics databases to constitute targets for negative regulation by miR-132-3p. These genes include *AKT3, NMT2, SIRT1* and *hTERT*. *AKT3* is a member of the AKT/PKB family of serine/threonine protein kinases. AKT3 regulates cell signaling events in response to insulin and IGF1 and has been linked to longevity control [58]. The *N*-myristoyltransferase-2 (*NMT2*) gene encodes an enzyme responsible for the addition of myristoyl groups to the *N*-terminal end of a number of proteins [59]. This lipid modification is associated with the cellular localization and function of signaling proteins. Both *AKT3* and *NMT2* genes were shown to be downregulated in gene expression experiments in LS vs control cells. However, we were unable to validate these results by means of qRT-PCR assays (Figure 3A).

Previous studies suggested that the *SIRT1* gene constitutes a potential target for miR-132-3p regulation and, furthermore, identified a functional correlation between *SIRT1* and miR-132-3p with potential relevance in human longevity [57]. In view of the elevated expression of miR-132-3p in LS, we assessed the expression of *SIRT1* in this condition by qRT-PCR. Results obtained revealed a 36% reduction in *SIRT1* mRNA levels in LS-derived lymphoblastoid cells (*p* < 0.05; Figure 3A).

Telomerase is a ribonucleoprotein polymerase responsible for adding the telomere repeat TTAGGG to chromosome ends. The protein component of telomerase, termed hTERT, displays reverse transcriptase activity while the RNA component of telomerase serves as a template for the telomere repeat. In view of the involvement of hTERT in senescence, we measured hTERT mRNA levels in LS cells. qRT-PCR revealed that hTERT mRNA levels were reduced by 54% compared to controls (Figure 3A).

### 2.4. Validation of miR-132-3p Target Genes by miR Manipulation

To directly assess the role of miR-132-3p in the regulation of *AKT3, NMT2, SIRT1* and *hTERT* mRNA expression, we employed the *mir*Vana™ system, consisting of miR-132-3p mimics and inhibitor in order to overexpress or silence, respectively, miR-132-3p in HEK293T cells. Effective expression or silencing was visualized by transient cotransfection of the LSB hsa-miR-132-3p plasmid, as described in the Materials and Methods. Extensive calibration experiments identified the optimal doses and incubation times for these experiments (data not shown). As shown in Figure 3B, transfection of miR-132-3p mimics led to marked (~1200-fold) overexpression of miR-132-3p after 24 h. On the other hand, transfection of miR-132-3p inhibitor led to drastic silencing of miR-132-3p expression.

Transfection of miR-132-3p mimics or inhibitor had a minor effect on *AKT3* gene expression (Figure 3A). Upregulation of miR-132-3p led to a small, but consistent, enhancement of *NMT2* expression (118%) whereas down-regulation of miR-132-3p was associated with a highly significant decrease in *NMT2* expression (39%) (*p* = 0.0001). An opposite pattern of regulation was seen in the case of *SIRT1*. Thus, addition of miR-132-3p mimics led to a 33% reduction in SIRT1 mRNA levels (*p* = 0.0197) whereas miR-132-3p inhibitor enhanced *SIRT1* gene expression by 146% (*p* = 0.0001). Finally, a direct correlation was seen between miR-132-3p and *hTERT* expression. The two-tailed *p*-value equals 0.0001 for mimics and 0.0339 for inhibitor.

### 2.5. Regulation of SIRT1 and hTERT Protein Levels by miR-132-3p Mimics and Inhibitor

**Figure 4:**
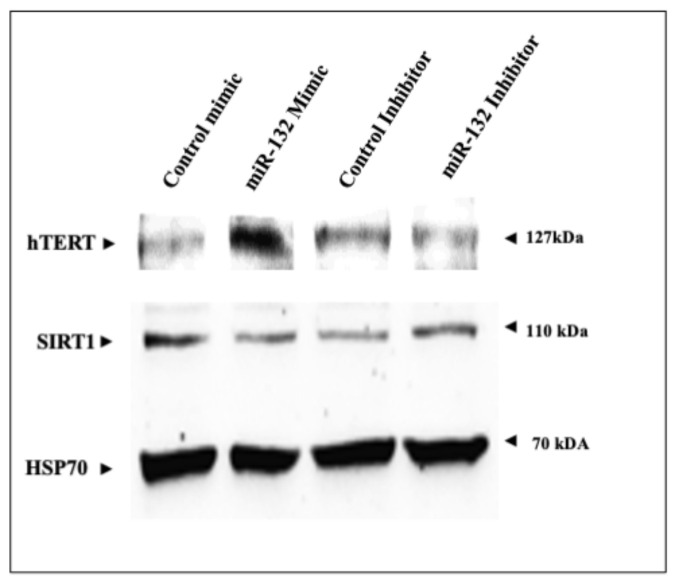
Western blot analysis of miR-132-3p effects on SIRT1 and hTERT levels. HEK293T cells were transfected with miR-132-3p mimics or inhibitor (or respective control) for 24 h, after which, hTERT and SIRT1 levels were measured by Western blot. Hsp70 was used as a loading control.

To establish whether changes in *SIRT1* and *hTERT* mRNA levels detected by qRT-PCR were correlated with parallel changes in protein levels, HEK293T cells were transfected with miR-132-3p mimics or inhibitor for 24 h, after which, total protein was isolated and SIRT1 and hTERT levels were measured by Western blots. Results obtained indicate that SIRT1 levels in mimics-transfected cells were lower than in control cells (Figure 4). On the other hand, SIRT1 levels were higher in miR-132-3p inhibitor-transfected cells. hTERT expression levels in miR-132-3p mimic-transfected cells were higher than in controls, while levels in miR-132-3p inhibitor-transfected cells were lower than in controls. These results indicate that changes in SIRT1 and hTERT protein levels upon miR-132-3p manipulation correlate with SIRT1 and hTERT mRNA expression.

### 2.6. Effect of miR-132-3p on Cell Cycle Progression

**Figure 5:**
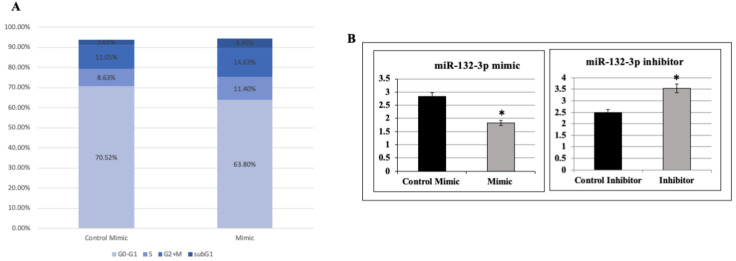
Effect of miR-132-3p on cell cycle progression and proliferation. (**A**) HEK293T cells were transfected with miR-132-3p mimics for 24 h, after which, the proportion of cells in each phase was measured by FACS analysis. (**B**) Cells were transfected with miR-132-3p mimics or inhibitor for 24 h, after which, cell number was assessed using a Cell Counter. * *p* < 0.01 versus control cells.

Next, experiments were carried out aimed at evaluating the impact of miR-132-3p on cell cycle progression. To this end, HEK293T cells were transfected with *mir*Vana™ miR-132-3p mimics, after which, the distribution of cells in the different phases of the cell cycle was analyzed by flow cytometry (Figure 5A). The proportion of cells in the G0/G1 phase was substantially decreased in the mimics group (from 70.52% in control cells to 63.80% in mimic-treated cells). Concomitantly, the proportion of cells in the S phase was increased (from 8.63% in control cells to 11.4% in mimic-treated cells). These data suggest that miR-132-3p mimic-treated cells moved from the G0/G1 phase (7% less cells) towards the S phase (3% more cells) compared to the control. Taken together, these results indicate that miR-132-3p might be involved in the regulation of cell cycle progression through G0/G1 to S transition.

### 2.7. Effect of miR-132-3p on Cell Proliferation

Next, we investigated the impact of miR-132-3p on cell proliferation. For this purpose, cells were transfected with *mir*Vana™ miR-132-3p mimics or inhibitor and, after 24 h, cell number was assessed using a Cell Counter. Results indicate that miR-132-3p mimic transfection led to a 36% reduction in cell proliferation whereas miR-132-3p inhibitor transfection led to a 142% increase in cell number (*p* < 0.05 versus controls; Figure 5B).

### 2.8. Correlations between IGF1, miR-132-3p and SIRT1

**Figure 6:**
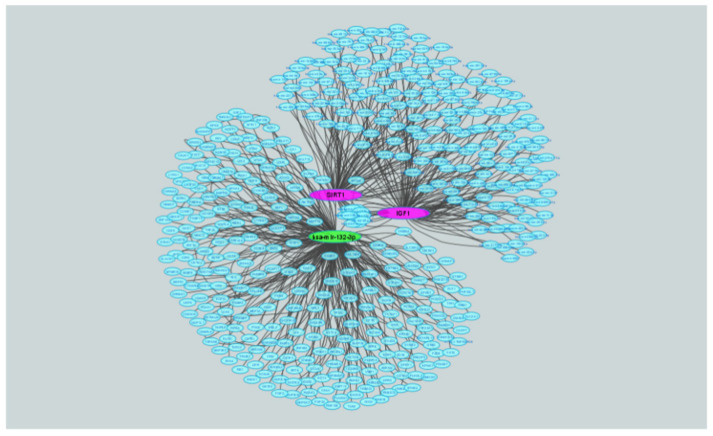
Graphic representation of the IGF1, miR-132-3p-SIRT1 loop. Network visual analysis of predicted targets of miR-132-3p and two key genes, SIRT1 and IGF1. The analysis identified 1668 entries of miR-gene interactions, which are presented using Cytoscape v. 3.7.2. miR-132-3p is presented in green and SIRT1 and IGF1 are presented in pink. All other interactions are shown in smaller light blue ellipses.

Finally, to represent in a graphic manner the functional and, potentially, physical correlation between IGF1, miR-132-3p and SIRT1, we conducted a network visual analysis. miR-132-3p, SIRT1 and IGF1 were analyzed by miRNet V.2.0 (https://www.mirnet.ca/miRNet/upload/MultiUploadView.xhtml, accessed on 15 July 2021) to decipher predicted targets using the miRTarBase 8.0 miR-gene database. The analysis revealed 1668 entries of miR-gene interactions, which were presented using Cytoscape v. 3.7.2. miR-132-3p is presented in green and SIRT1 and IGF1 are presented in pink. All other interactions are shown in smaller light blue ellipses (Figure 6).

## 3. Discussion

The GH/IGF1 endocrine system plays key roles in metabolism and growth regulation [2]. Deregulated expression of components of this axis has been linked to a number of pathologies, including cancer [1,60]. Congenital IGF1 deficiencies provide a unique opportunity to investigate multiple aspects of the GH/IGF1 system and, despite the rarity of these conditions, important biological paradigms were derived from these ‘*experiments of nature*’ [7,8,61]. The Laron syndrome constitutes the best characterized entity under the umbrella of the IGF1 pathologies [21]. Comprehensive analyses of this disease over half a century have had a major impact on our current understanding of normal and aberrant growth [43,62,63].

The epidemiological evidence that LS patients are protected from cancer development is of major basic and clinical relevance [28,29]. This finding is consistent with the concept that the GH/IGF1 signaling system has a fundamental role in the cell’s decision of whether to adopt proliferative or apoptotic paths [64,65,66,67]. While cancer and aging are usually considered radically opposite processes, new lines of evidence provide support to the notion that cancer and aging might, in fact, be regarded as different manifestations of the same underlying biochemical and cellular processes [68]. These processes include accumulation of cellular damage, genomic instability, epigenetic alterations, deregulated nutrient sensing, mitochondrial dysfunction and others [69].

The present study identified miR-132-3p as a highly represented microRNA in LS. Given the fact that the typical hallmark of LS is a drastic reduction in circulating IGF1 levels, the data is consistent with the postulate that miR-132-3p constitutes a target for inhibitory regulation by IGF1. While the mechanistic aspects of this novel regulatory loop are yet to be investigated, our bioinformatics analyses—based on miR and gene expression experiments—have identified a series of genes whose expression is directly regulated by miR-132-3p. MicroRNA target databases are often based on prediction and, usually, are highly noisy. In addition, they are not specific for a given tissue or condition. Therefore, in order to present more specific data, we performed our bioinformatics analyses—based on miR and gene expression—on the same LS and control human donor samples. Hence, these experiments enabled correlation analysis between gene and miR experimental data performed under similar conditions.

miR-132-3p has been linked to a number of human diseases and is being pursued as a potential clinical biomarker [70,71]. Several targets for miR-132-3p have been described, including mediators of neurological development, synaptic transmission, inflammation and angiogenesis [56,72]. In the nervous system, miR-132-3p is involved in the regulation of neuronal differentiation, maturation and functioning, and also participates in axon growth, neuronal migration and plasticity [73]. A recent study demonstrated that the miR-132/212 network contributes to abnormal Aβ metabolism and senile plaque deposition in Alzheimer’s disease (AD) [74]. Among other genes shown to be regulated by miR-132-3p, our analyses identified SIRT1 as a biologically relevant target for negative miR-132-3p control. These results are in agreement with those reported by Hadar et al. [57], showing a four-fold lower expression of SIRT1 and a correspondingly higher expression of miR-132 in lymphoblastoid cells derived from AD patients compared to healthy controls. In addition, correlations of SIRT1 and miR-132 with cognitive scores were observed.

SIRT1 is the most extensively studied member of the sirtuins, a family of mammalian class III histone deacetylases implicated in health span and longevity. SIRT1 has multiple physiological functions as well as wide-ranging roles in pathological settings [75,76]. SIRT1 is one of seven human sirtuins that possess mono-ADP-ribosyltransferase or deacetylase activity toward target proteins, including histones. SIRT1 regulates endocrine, mitochondrial, circadian rhythm and hypothalamic functions [77,78]. In the brain, SIRT1 takes part in memory formation by modulating synaptic plasticity, and promoting axonal elongation and dendritic branching [79]. Among the seven mammalian sirtuins, SIRT1 is best recognized as being associated with longevity and neuroprotection. The identification of SIRT1 as a *bona fide* target for miR-132-3p provides a physical foundation to the observation that disruption of the GH/IGF1 axis correlates with enhanced lifespan [80].

The question of whether the physiological decrease in GH and IGF1 levels with advanced age affects lifespan has been a topic of major interest for many years [81,82,83]. A recent study examined prospective associations of serum IGF1 with mortality, dementia, vascular disease, diabetes, osteoporosis and cancer—finding two generalized patterns [84]. First, younger individuals with high IGF1 are protected from disease while older individuals with high IGF1 are at enhanced risk for incident disease or death. Second, the association between IGF1 and disease risk is U-shaped, suggesting that both high and low IGF1 dosages might be unfavorable. A remarkable exception to this pattern is the case of cancer, which is typically associated with a positive correlation with IGF1 levels. Taken together, these analyses suggest that IGF1 signaling could be harmful in older adults. Untreated patients with LS constitute a unique model for testing the impact of low IGF1 on lifespan and healthspan [36]. However, LS patients are spread worldwide and, with the exception of a very small number of cohorts, are dispersed among many physicians, and if not treated as children, are lost to follow-up [21]. Present knowledge shows that lifelong IGF1 deficiency in untreated patients with LS does not seem to markedly influence their lifespan, unless they neglect to treat in time the metabolic and cardiovascular complications of this condition, such as diabetes mellitus and hyperlipidemia.

Despite a lack of conclusive epidemiological evidence on longevity in LS and other congenital IGF1 deficiencies, the importance of GH/IGF1 signaling in the control of lifespan was clearly established by the demonstration that mice lacking GH or GHRs live much longer than their normal siblings [85]. In GHR-KO (‘*Laron*’) mice lifespan extension was shown in four independent studies using animals of three different genetic backgrounds and diets of different macronutrient compositions [86,87]. Thus, female ‘*Laron*’ mice have a 38% longer lifespan than wild type animals, while male mice have a 55% extended lifespan. Disruption of the insulin/IGF1 signaling pathway was similarly associated with extended lifespan in various lower species, including *D. melanogaster* and *C. elegans* [66,88].

In conclusion, we have identified miR-132-3p as a highly expressed miR in LS. Data reveal that low endocrine levels of IGF1 in this condition lead to elevated miR-132-3p levels, with ensuing reductions in SIRT1—and, probably, additional gene expression. The transcriptional and epigenetic mechanisms responsible for the concerted expression and activity of the IGF1-miR-132-3p-SIRT1 axis are yet to be dissected. Finally, the impact of this regulatory loop on longevity merits further investigation.

## 4. Materials and Methods

### 4.1. Cell Cultures

Epstein–Bar virus (EBV)-immortalized lymphoblastoid cell lines from LS patients and healthy controls of the same age range, gender and ethnic group were obtained from the National Laboratory for the Genetics of Israeli Populations (NLGIP; http://yoran.tau.ac.il/nlgip/, accessed on 1 March 2020), Tel Aviv University, Israel. All immortalized lymphoblastoid cell lines were generated from peripheral blood B lymphocytes donated by consenting individuals. Of note, a written informed consent form was obtained from all donors. Cells were maintained in RPMI-1640 medium supplemented with 10% fetal bovine serum (FBS), 2 mM glutamine and antibiotics. Reagents were purchased from Biological Industries Ltd. (Kibutz Beit-Haemek, Israel). Cultures were grown upright in tissue culture flasks and maintained under a humidified 5% CO_2_ atmosphere at 37 °C. Human embryonic kidney cells (HEK293T) were maintained in DMEM medium supplemented with 10% FBS, 2 mM glutamine and antibiotics.

### 4.2. Affymetrix GeneChip MicroRNA Analyses of LS-Derived Cells

GeneChip^®^ miRNA 4.0 arrays (Affymetrix, CA, USA), which contain the entire repertoire of currently known small ncRNA transcripts involved in gene expression (30,434 total mature miRNA probe sets for 203 organisms, including 2578 human miRNAs) were used for genome-wide miRNA-expression analysis. Total RNA was prepared as described below and miR expression was analyzed at the Center for Genomic Technologies, Hebrew University of Jerusalem, Israel.

### 4.3. Bioinformatics Analysis

CEL files were loaded to Partek® Genomics Suite software (https://www.partek.com/partek-genomics-ssuite/, accessed on 15 July 2021) and analyzed with RMA [89]; background correction was normalized by Quantile normalization [90,91]. Log2 expression data was obtained and Probeset summarization performed by Median Polish [92]. ANOVA was performed and 68 differently expressed human miRs were identified between LS and controls (cutoff *p* < 0.05 and fold-change difference = 1.5).

### 4.4. Quantitative Real Time-Polymerase Chain Reaction for MicroRNA Expression Analysis

Total RNA was prepared from cells using the Trizol reagent (ThermoFisher Scientific, Waltham, MA, USA). Two-hundred ng of total RNA was reverse transcribed using the Superscript First-Strand Synthesis System for cDNA synthesis by PCR (ThermoFisher Scientific). Quantitative real-time PCR SYBR Green qRT-PCR was performed using 200 nM of each PerfeCTa^®^ microRNA Assay Primer and PerfeCTa^®^ Universal PCR Primer, along with the appropriate PerfeCTa^®^ SYBR Green SuperMix (ThermoFisher Scientific). For each qRT-PCR reaction, the following components were added: PerfeCTa^®^ SYBR Green SuperMix, PerfeCTa^®^ microRNA Assay Primer (10 μM) and PerfeCTa^®^ Universal PCR Primer (10 μM). miR-132-3p, miR-200c-3p, miR-362-5p and miR-421 levels were measured by qRT-PCR, using appropriate primers. For control purposes, levels of PerfeCTa^®^ Human Positive Control Primer were measured.

### 4.5. qRT-PCR for Gene Expression Analysis

SIRT1, AKT3, hTERT and NMT2 mRNA levels were measured by qRT-PCR using FastStart Universal SYBR Green (Rox, Roche Diagnostics GmBH, Mannheim, Germany), with appropriate primers. For control purposes, levels of β-actin mRNA were measured. Amplifications were carried out after an incubation of 2 min at 50 °C and 10 min at 95 °C, followed by 40 cycles at 95 °C for 15 s, 1 min at 55 °C, and 30 s at 72 °C. The number of PCR cycles to reach the fluorescence threshold is the cycle threshold (Ct). Each cDNA sample was tested in triplicate and mean Ct values are reported. For each reaction, a “no template” sample was included as a negative control. Fold-differences were calculated using the 2ΔΔCt method [93]. An ABI Prism 7000 Sequence Detection System was employed for detection.

### 4.6. MiR-132-3p Inhibitor and Mimics Experiments

*mir*Vana™ miRNA inhibitor and mimics of miR-132-3p were purchased from ThermoFisher Scientific and optimized by dividing into two groups: with and without treatment. Optimizations were performed by seeding 2.5 × 10^5^ HEK293T cells into 6-well plates for 24, 48, 72 or 96 h. The Low Sensor Backbone (LSB)-hsa-miR-132-3p plasmid (Addgene, Watertown, MA, USA; Cat. number #103219) was used to sense miR activity using fluorescence-based tools. The plasmid encodes constitutive expression of two fluorescent proteins from the hEF1a promoter, one of which (mKate2) is regulated by miRNA target sites in the 3′ UTR while the other (EBFP2) serves as a transfection marker. Target sites consist of four repeats of the sequence complementary to the mature human miRNA sequence. The plasmid was transfected into HEK293T cells according to the supplier’s protocol. miRNAs with detectable activity downregulated mKate2 fluorescence relative to EBFP2, which was measured using fluorescence microscopy.

### 4.7. Western Blot Analysis

Cells were harvested with ice-cold phosphate-buffered saline (PBS) (Biological Industries Ltd) containing 5 mM EDTA and centrifuged at 1100 rpm. Lysis buffer was added to the cells (200 μL per 5 × 10^6^ cells) and they were incubated on ice for 10 min. After incubation, cells were centrifuged at 13,000 rpm for 10 min. Protein concentration was determined by the Bradford method (Bio-Rad Laboratories Ltd., Hercules, CA, USA). Similar volumes of each sample were divided into 96-well microplates. Optical density was measured using a microplate reader. Lysates were electrophoresed through 10% SDS-PAGE and then blotted onto nitrocellulose membranes. After blocking, the blots were incubated overnight with the indicated antibodies, washed and incubated with a horseradish peroxidase-conjugated secondary antibody.

### 4.8. Cell Cycle Analysis

Twenty-four hours prior to miR-132-3p manipulation, cells were split into 6-well plates (2.5 × 10^5^ cells/well). After 6 h, cells were transfected with *mir*Vana™ inhibitor or mimic, as described above. After an additional 24 h (according to results from optimization experiments), cells were washed with PBS, trypsinized, permeabilized with Triton X-100 (4%) and stained with propidium iodide. Stained cells were analyzed using a FACSort flow cytometer (Becton Dickinson, Franklin Lakes, NJ, USA).

### 4.9. Cell Proliferation Assays

Cells were counted in an automated cell counter (CelloMeter, Beckman Coulter Life Sciences, Indianapolis, IN, USA). In addition, cell proliferation was assessed using an XTT cell proliferation kit (Biological Industries Ltd.) according to the manufacturer’s instructions. Twenty-four hours post-inhibition or mimic transfection, cells were seeded into 96-well plates (1 × 10^4^ cells/well) and, after an additional 24 h, the XTT reagent was added. One–two hours after addition of the XTT reagent, sample absorbance was measured with a spectrophotometer at a wavelength of 450–500 nanometers.

### 4.10. Statistical Analysis

The statistical significance of the differences between groups was assessed by Student’s t-test (two samples, equal variance). Data are presented as mean ± SEM of two or three independent experiments. *p* values < 0.05 were considered statistically significant.

## Figures and Tables

**Table 1:** Sixty-eight human miRs were differentially expressed in LS vs Control (FC = 1.5 and *p* < 0.05). Data was combined with the 130 differentially expressed genes from the mRNA analysis (same cutoff). Only miR-gene targets that were negatively correlated were selected. First three rows represent miR information, including miR name, *p*-value and fold-change in LS vs. control (up-regulation in pink and down-regulation in gray). The additional columns present miR-targets from gene expression experiments, including *p*-value and fold-change as revealed above. Pearson’s correlation analysis presents negative correlations between miR and gene fold-change differences with significant *p*-values (last column).

Transcript ID (Array Design)	*p*-Value (LS vs. Con)	Fold-Change (LS vs. Con)	Gene_Assignment	Gene Symbol	RefSeq	*p*-Value (LS vs. Con)	Fold-Change (LS vs. Con)	Pearson’s Correlation	*p*-Value
hsa-miR-132-3p	0.027634	3.20613	AY005799 // AKT3 // v-akt murine thymoma viral oncogene homolog 3 (protein kinase B, ga)	AKT3	AY005799	0.034986	−1.51126	−0.76	0.027
hsa-miR-132-3p	0.027634	3.20613	AF043325 // NMT2 // N-myristoyltransferase 2	NMT2	AF043325	0.007468	−1.53421	−0.76	0.027
hsa-miR-362-5p	0.031091	2.43203	AY005799 // AKT3 // v-akt murine thymoma viral oncogene homolog 3 (protein kinase B, ga)	AKT3	AY005799	0.034986	−1.51126	−0.75	0.031
hsa-miR-421	0.027239	2.39773	AB015706 // IL6ST // interleukin 6 signal transducer (gp130, oncostatin M receptor)	IL6ST	AB015706	0.022031	−1.63661	−0.76	0.027
hsa-miR-421	0.027239	2.39773	BC035128 // MXI1 // MAX interactor 1 // 10q24-q25	MXI1	BC035128	0.005779	−1.62721	−0.76	0.027
hsa-miR-200c-3p	0.047791	2.00074	AB015706 // IL6ST // interleukin 6 signal transducer (gp130, oncostatin M receptor)	IL6ST	AB015706	0.022031	−1.63661	−0.71	0.047
hsa-miR-200c-3p	0.047791	2.00074	BC035128 // MXI1 // MAX interactor 1 // 10q24-q25	MXI1	BC035128	0.005779	−1.62721	−0.71	0.047
hsa-miR-200c-3p	0.047791	2.00074	AK302477 // NPNT // nephronectin // 4q24 // 255743	NPNT	AK302477	0.013197	−3.14456	−0.71	0.047
hsa-miR-200c-3p	0.047791	2.00074	BC000261 // THRA // thyroid hormone receptor, alpha	THRA	BC000261	0.007158	−1.52116	−0.71	0.047
hsa-miR-532-5p	0.029735	1.65873	AB015706 // IL6ST // interleukin 6 signal transducer (gp130, oncostatin M receptor)	IL6ST	AB015706	0.022031	−1.63661	−0.75	0.029
hsa-miR-532-5p	0.029735	1.65873	AF252872 // SLC7A11 // solute carrier family 7 (anionic amino acid transporter light ch)	SLC7A11	AF252872	0.023959	−1.632	−0.75	0.029
hsa-miR-199a-3p	0.033323	−1.58718	AB073146 // DCBLD2 // discoidin, CUB and LCCL domain containing 2	DCBLD2	AB073146	0.00852	1.58136	−0.74	0.033
hsa-miR-30b-5p	0.032193	−1.79613	AF091433 // CCNE2 // cyclin E2 // 8q22.1 // 9134	CCNE2	AF091433	0.000984	1.55079	−0.74	0.032
hsa-miR-30b-5p	0.032193	−1.79613	BC017338 // FUCA1 // fucosidase, alpha-L- 1, tissue	FUCA1	BC017338	0.00248	2.34712	−0.74	0.032
hsa-miR-30b-5p	0.032193	−1.79613	AB039723 // FZD3 // frizzled family receptor 3 // 8p21	FZD3	AB039723	0.014296	1.59799	−0.75	0.032
hsa-miR-30b-5p	0.032193	−1.79613	AF186780 // RGL1 // ral guanine nucleotide dissociation stimulator-like 1	RGL1	AF186780	0.037572	1.8744	−0.75	0.032
hsa-miR-30b-5p	0.032193	−1.79613	AF111801 // TRERF1 // transcriptional regulating factor 1	TRERF1	AF111801	0.041722	1.9583	Not in table	
hsa-miR-30b-5p	0.032193	−1.79613	AM404182 // TRERF1 // transcriptional regulating factor	TRERF1	AM404182	0.036958	1.71707	Not in table	

## References

[1] Yakar S., Adamo M.L. (2012). Insulin-like growth factor 1 physiology: Lessons from mouse models. Endocrinol. Metab. Clin. N. Am..

[2] LeRoith D., Yakar S. (2007). Mechanisms of disease: Metabolic effects of growth hormone and insulin-like growth factor-1. Nat. Clin. Pract. Endocrinol. Metab..

[3] Bondy C.A., Werner H., Roberts C.T., LeRoith D. (1990). Cellular pattern of insulin-like growth factor I (IGF-I) and type I IGF receptor gene expression in early organogenesis: Comparison with IGF-II gene expression. Mol. Endocrinol..

[4] Werner H., Woloschak M., Adamo M., Shen-Orr Z., Roberts C.T., LeRoith D. (1989). Developmental regulation of the rat insulin-like growth factor I receptor gene. Proc. Natl. Acad. Sci. USA.

[5] Yakar S., Werner H., Rosen C.J. (2018). Insulin-like growth factors: Actions on the skeleton. J. Mol. Endocrinol.

[6] Salmon W.D., Daughaday W.H. (1957). A hormonally controlled serum factor which stimulates sulfate incorporation by cartilage in vitro. J. Lab. Clin. Med..

[7] Rosenfeld R.G. (2003). Insulin-like growth factors and the basis of growth. N. Engl. J. Med..

[8] Domené S., Domené H.M. (2018). Genetic mutations in the GH/IGF axis. Pediatr. Endocrinol. Rev..

[9] Renehan A.G., Frystyk J., Flyvbjerg A. (2006). Obesity and cancer risk: The role of the insulin-IGF axis. Trends Endocrinol. Metab..

[10] Chan J.M., Stampfer M.J., Giovannucci E., Gann P.H., Ma J., Wilkinson P., Hennekens C.H., Pollak M. (1998). Plasma insulin-like growth factor-I and prostate cancer risk: A prospective study. Science.

[11] Hankinson S.E., Willett W.C., Colditz G.A., Hunter D.J., Michaud D.S., Deroo B., Rosner B., Speizer F.E., Pollak M. (1998). Circulating concentrations of insulin-like growth factor-I and risk of breast cancer. Lancet.

[12] Kaaks R., Lukanova A. (2001). Energy balance and cancer: The role of insulin and insulin-like growth factors. Proc. Nutr. Soc..

[13] Werner H., Bruchim I. (2012). IGF-1 and BRCA1 signalling pathways in familial cancer. Lancet Oncol..

[14] Sarfstein R., Maor S., Reizner N., Abramovitch S., Werner H. (2006). Transcriptional regulation of the insulin-like growth factor-1 receptor in breast cancer. Mol. Cell. Endocrinol..

[15] Pollak M. (2012). The insulin and insulin-like growth factor receptor family in neoplasia: An update. Nat. Rev. Cancer.

[16] Werner H. (2012). Tumor suppressors govern insulin-like growth factor signaling pathways: Implications in metabolism and cancer. Oncogene.

[17] Baserga R. (2000). The contradictions of the insulin-like growth factor 1 receptor. Oncogene.

[18] Holly J.M., Perks C.M. (2012). Insulin-like growth factor physiology: What we have learned from human studies. Endocrinol. Metab. Clin. North Am..

[19] LeRoith D. (2008). Clinical relevance of systemic and local IGF-I: Lessons from animal models. Pediatr. Endocrinol. Rev..

[20] Laron Z. (2004). Extensive personal experience. Laron syndrome (primary growth hormone resistance or insensitivity): The personal experience 1958-2003. J. Clin. Endocrinol. Metab..

[21] Laron Z., Kopchik J.J. (2011). Laron Syndrome—From Man to Mouse.

[22] Laron Z., Pertzelan A., Mannheimer S. (1966). Genetic pituitary dwarfism with high serum concentration of growth hormone-a new inborn error of metabolism?. Isr. J. Med. Sci..

[23] Eshet R., Werner H., Klinger B., Silbergeld A., Laron Z., LeRoith D., Roberts C.T. (1993). Up-regulation of insulin-like growth factor-I (IGF-I) receptor gene expression in patients with reduced serum IGF-I levels. J. Mol. Endocrinol..

[24] Shevah O., Laron Z. (2006). Genetic analysis of the pedigrees and molecular defects of the GH-receptor gene in the Israeli cohort of patients with Laron syndrome. Pediatr. Endocrinol. Rev..

[25] Amselem S., Duquesnoy P., Attree O., Novelli G., Bousnina S., Postel-Vinay M.C., Goossens M. (1989). Laron dwarfism and mutations of the growth hormone-receptor gene. N. Engl. J. Med..

[26] Godowski P.J., Leung D.W., Meacham L.R., Galgani J.P., Hellmiss R., Keret R., Rotwein P.S., Parks J.S., Laron Z., Wood W.I. (1989). Characterization of the human growth hormone receptor gene and demonstration of a partial gene deletion in two patients with Laron-type dwarfism. Proc. Natl. Acad. Sci. USA.

[27] Shevah O., Laron Z. (2007). Patients with congenital deficiency of IGF-I seem protected from the development of malignancies: A preliminary report. Growth Horm. IGF Res..

[28] Steuerman R., Shevah O., Laron Z. (2011). Congenital IGF1 deficiency tends to confer protection against post-natal development of malignancies. Eur. J. Endocrinol..

[29] Guevara-Aguirre J., Balasubramanian P., Guevara-Aguirre M., Wei M., Madia F., Cheng C.W., Hwang D., Martin-Montalvo A., Saavedra J., Ingles S. (2011). Growth hormone receptor deficiency is associated with a major reduction in pro-aging signaling, cancer, and diabetes in humans. Sci. Transl. Med..

[30] Vitale G., Pellegrino G., Vollery M., Hofland L.J. (2019). Role of IGF-1 system in the modulation of longevity: Controversies and new insights from a centenarian’s perspective. Front. Endocrinol..

[31] Kimura K.D., Tissenbaum H.A., Liu Y., Ruvkun G. (1997). daf-2, an insulin receptor-like gene that regulates longevity and diapause in Caenorhabditis elegans. Science.

[32] Tazearslan C., Huang J., Barzilai N., Suh Y. (2011). Impaired IGF1R signaling in cells expressing longevity-associated human IGF1R alleles. Aging Cell.

[33] Coschigano K.T., Clemmons D., Bellush L.L., Kopchick J.J. (2000). Assessment of growth parameters and life span of GHR/BP gene-disrupted mice. Endocrinology.

[34] Basu R., Qian Y., Kopchick J.J. (2018). Lessons from growth hormone receptor gene-disrupted mice: Are there benefits of endocrine defects?. Eur. J. Endocrinol..

[35] Carter C.S., Ramsey M.M., Sonntag W.E. (2002). A critical analysis of the role of growth hormone and IGF-1 in aging and lifespan. Trends Genet..

[36] Laron Z., Kauli R., Lapkina L., Werner H. (2017). IGF-I deficiency, longevity and cancer protection of patients with Laron syndrome. Mutat. Res. Rev. Mutat. Res..

[37] Bartke A. (2003). Can growth hormone (GH) accelerate aging? Evidence from GH-transgenic mice. Neuroendocrinology.

[38] Lapkina-Gendler L., Rotem I., Pasmanik-Chor M., Gurwitz D., Sarfstein R., Laron Z., Werner H. (2016). Identification of signaling pathways associated with cancer protection in Laron syndrome. Endocr. Relat. Cancer.

[39] Nagaraj K., Lapkina-Gendler L., Sarfstein R., Gurwitz D., Pasmanik-Chor M., Laron Z., Yakar S., Werner H. (2018). Identification of thioredoxin-interacting protein (TXNIP) as a downstream target for IGF1 action. Proc. Natl. Acad. Sci. USA.

[40] Achlaug L., Sarfstein R., Nagaraj K., Lapkina-Gendler L., Bruchim I., Dixit M., Laron Z., Yakar S., Werner H. (2019). Identification of ZYG11A as a candidate IGF1-dependent proto-oncogene in endometrial cancer. Oncotarget.

[41] Sarfstein R., Lapkina-Gendler L., Nagaraj K., Laron Z., Werner H. (2020). Identification of nephronectin as a new target for IGF1 action. Eur. J. Cancer.

[42] Shibel R., Sarfstein R., Nagaraj K., Lapkina-Gendler L., Laron Z., Dixit M., Yakar S., Werner H. (2021). The olfactory receptor gene product, OR5H2, modulates endometrial cancer cells proliferation via interaction with the IGF1 signaling pathway. Cells.

[43] Werner H., Sarfstein R., Nagaraj K., Laron Z. (2020). Laron syndrome research paves the way for new insights in oncological investigation. Cells.

[44] Guarnieri D.J., DiLeone R.J. (2008). MicroRNAs: A new class of gene regulators. Ann. Med..

[45] Zalts H., Shomron N. (2011). The impact of microRNAs on endocrinology. Pediatr. Endocrinol. Rev..

[46] Hobert O. (2008). Gene regulation by transcription factors and microRNAs. Science.

[47] Hornstein E., Shomron N. (2006). Canalization of development by microRNAs. Nat. Genet..

[48] Nana-Sinkam S.P., Croce C.M. (2011). MicroRNAs as therapeutic targets in cancer. Transl. Res..

[49] Jiang L., Liu X., Chen Z., Jin Y., Heidbreder C.E., Kolokythas A., Wang A., Dai Y., Zhou X. (2010). MicroRNA-7 targets IGF1R (insulin-like growth factor 1 receptor) in tongue squamous cell carcinoma cells. Biochem J..

[50] Shu S., Liu X., Xu M., Gao X., Chen S., Zhang L., Li R. (2019). MicroRNA-320a acts as a tumor suppressor in endometrial carcinoma by targeting IGF-1R. Int. J. Mol. Med..

[51] McKinsey E.L., Parrish J.K., Irwin A.E., Niemeyer B.F., Kern H.B., Birks D.K., Jedlicka P. (2011). A novel oncogenic mechanism in Ewing sarcoma involving IGF pathway targeting by EWS/Fli1-regulated microRNAs. Oncogene.

[52] Dobre M., Herlea V., Vladut C., Ciocirlan M., Balaban V.D., Constantinescu G., Diculescu M., Milanesi E. (2021). Dysregulation of miRNAs targeting the IGF-1R pathway in pancreatic ductal adenocarcinoma. Cells.

[53] Catellani C., Ravegnini G., Sartori C., Angelini S., Street M.E. (2021). GH and IGF system: The regulatory role of miRNAs and lncRNAs in cancer. Front. Endocrinol..

[54] Cirillo F., Catellani C., Lazzeroni P., Sartori C., Street M.E. (2020). The role of microRNAs in influencing body growth and development. Horm. Res. Paediatr..

[55] Anand S., Majeti B.K., Acevedo L.M., Murphy E.A., Mukthavaram R., Scheppke L., Huang M., Shields D.J., Lindquist J.N., Lapinski P.E. (2010). MicroRNA-132-mediated loss of p120RasGAP activates the endothelium to facilitate pathological angiogenesis. Nat. Med..

[56] Li D., Wang A., Liu X., Meisgen F., Grünler J., Botusan I.R., Narayanan S., Erikci E., Li X., Blomqvist L. (2015). MicroRNA-132 enhances transition from inflammation to proliferation during wound healing. J. Clin. Investig..

[57] Hadar A., Milanesi E., Walczak M., Puzianowska-Kuźnicka M., Kuźnicki J., Squassina A., Niola P., Chillotti C., Attems J., Gozes I. (2018). SIRT1, miR-132 and miR-212 link human longevity to Alzheimer’s Disease. Sci. Rep..

[58] Deelen J., Uh H.W., Monajemi R., van Heemst D., Thijssen P.E., Böhringer S., van den Akker E.B., de Craen A.J., Rivadeneira F., Uitterlinden A.G. (2013). Gene set analysis of GWAS data for human longevity highlights the relevance of the insulin/IGF-1 signaling and telomere maintenance pathways. Age.

[59] Mackey J.R., Lai J., Chauhan U., Beauchamp E., Dong W.F., Glubrecht D., Sim Y.W., Ghosh S., Bigras G., Lai R. (2021). N-myristoyltransferase proteins in breast cancer: Prognostic relevance and validation as a new drug target. Breast Cancer Res. Treat..

[60] Werner H., Laron Z. (2020). Role of the GH-IGF1 system in progression of cancer. Mol. Cell. Endocrinol..

[61] Klammt J., Pfaffle R., Werner H., Kiess W. (2008). IGF signaling defects as causes of growth failure and IUGR. Trends Endocrinol. Metab..

[62] Laron Z. (2015). Lessons from 50 years of study of Laron syndrome. Endocr. Pract..

[63] Werner H., Lapkina-Gendler L., Laron Z. (2017). Fifty years on: New lessons from Laron syndrome. Isr. Med. Assoc. J..

[64] Werner H., Sarfstein R. (2014). Transcriptional and epigenetic control of IGF1R gene expression: Implications in metabolism and cancer. Growth Horm. IGF Res..

[65] Baserga R. (2013). The decline and fall of the IGF-I receptor. J. Cell. Physiol..

[66] Kenyon C. (2001). A conserved regulatory system for aging. Cell.

[67] Juul A. (2003). Serum levels of insulin-like growth factor I and its binding proteins in health and disease. Growth Horm IGF Res..

[68] Anisimov V.N., Bartke A. (2013). The key role of growth hormone-insulin-IGF-1 signaling in aging and cancer. Crit. Rev. Oncol. Hematol..

[69] Lopez-Otin C., Blasco M.A., Partridge L., Serrano M., Kroemer G. (2013). The hallmarks of aging. Cell.

[70] Chen C., Lu C., Qian Y., Li H., Tan Y., Cai L., Weng H. (2017). Urinary miR-21 as a potential biomarker of hypertensive kidney injury and fibrosis. Sci. Rep..

[71] Akgör U., Ayaz L., Çayan F. (2021). Expression levels of maternal plasma microRNAs in preeclamptic pregnancies. J. Obstet. Gynecol..

[72] Kariba Y., Yoshizawa T., Sato Y., Tsuyama T., Araki E., Yamagata K. (2020). Brown adipocyte-derived exosomal miR-132-3p suppress hepatic Srebf1 expression and thereby attenuate expression of lipogenic genes. Biochem. Biophys. Res. Commun..

[73] Qian Y., Song J., Ouyang Y., Han Q., Chen W., Zhao X., Xie Y., Chen Y., Yuan W., Fan C. (2017). Advances in roles of miR-132 in the nervous system. Front. Pharmacol..

[74] Hernandez-Rapp J., Rainone S., Goupil C., Dorval V., Smith P.Y., Saint-Pierre M., Vallée M., Planel E., Droit A., Calon F. (2016). microRNA-132/212 deficiency enhances Aβ production and senile plaque deposition in Alzheimer’s disease triple transgenic mice. Sci. Rep..

[75] Tang B.L. (2016). Sirt1 and the mitochondria. Mol. Cells.

[76] Leite J.A., Ghirotto B., Targhetta V.P., de Lima J., Câmara N.O.S. (2021). Sirtuins as pharmacological targets in neurodegenerative and neuropsychiatric disorders. Br. J. Pharmacol..

[77] Strum J.C., Johnson J.H., Ward J., Xie H., Feild J., Hester A., Alford A., Waters K.M. (2009). MicroRNA 132 regulates nutritional stress-induced chemokine production through repression of SirT1. Mol. Endocrinol..

[78] Ye F., Wu A. (2021). The protective mechanism of SIRT1 in the regulation of mitochondrial biogenesis and mitochondrial autophagy in Alzheimer’s Disease. J. Alzheimers Dis..

[79] Mishra P., Mittal A.K., Kalonia H., Madan S., Ghosh S., Sinha J.K., Rajput S.K. (2021). SIRT1 promotes neuronal fortification in neurodegenerative diseases through attenuation of pathological hallmarks and enhancement of cellular lifespan. Curr. Neuropharmacol..

[80] Tran D., Bergholz J., Zhang H., He H., Wang Y., Zhang Y., Li Q., Kirkland J.L., Xiao Z.X. (2014). Insulin-like growth factor-1 regulates the SIRT1-p53 pathway in cellular senescence. Aging Cell.

[81] Burgers A.M.G., Biermasz N.R., Schoones J.W., Pereira A.M., Renehan A.G., Zwahlen M., Egger M., Dekkers O.M. (2011). Meta-analysis and dose-response metaregression: Circulating insulin-like growth factor I (IGF-I) and mortality. J. Clin. Endocrinol. Metab..

[82] Friedrich N., Haring R., Nauck M., Lüdemann J., Rosskopf D., Spilcke-Liss E., Felix S.B., Dörr M., Brabant G., Völzke H. (2009). Mortality and serum insulin-like growth factor (IGF)-I and IGF binding protein 3 concentrations. J. Clin. Endocrinol. Metab..

[83] Milman S., Huffman D.M., Barzilai N. (2016). The somatotropic axis in human aging: Framework for the current state of knowledge and future research. Cell Metab..

[84] Zhang W.B., Ye K., Barzilai N., Milman S. (2021). The antagonistic pleiotropy of insulin-like growth factor 1. Aging Cell.

[85] Aguiar-Oliveira M.H., Bartke A. (2019). Growth hormone deficiency: Health and longevity. Endocr. Rev..

[86] Bartke A. (2011). Pleiotropic effects of growth hormone signaling in aging. Trends Endocrinol. Metab..

[87] Bartke A., Sun L.Y., Longo V. (2013). Somatotropic signaling: Trade-offs between growth, reproductive development, and longevity. Physiol. Rev..

[88] Broughton S.J., Piper M.D.W., Ikeya T., Bass T.M., Jacobson J., Driege Y., Martinez P., Hafen E., Withers D.J., Leevers S.J. (2005). Longer lifespan, altered metabolism, and stress resistance in Drosophila from ablation of cells making insulin-like ligands. Proc. Natl. Acad. Sci. USA.

[89] Wu Z.S., Irizarry R.A., Gentleman R., Martines-Murillo F., Spencer F. (2004). A model-based background adjustment for oligonucleotide expression arrays. J. Am. Stat. Assoc..

[90] Bolstad B.M., Irizarry R.A., Astrand M., Speed T.P. (2003). A comparison of normalization methods for high density oligonucleotide array data based on variance and bias. Bioinformatics.

[91] Irizarry R.A., Hobbs B., Collin F., Beazer-Barclay Y.D., Antonellis K.J., Scherf U., Speed T.P. (2003). Exploration, normalization, and summaries of high density oligonucleotide array probe level data. Biostatistics.

[92] Irizarry R.A., Bolstad B.M., Collin F., Cope L.M., Hobbs B., Speed T.P. (2003). Summaries of Affymetrix GeneChip probe level data. Nucleic Acids Res..

[93] Livak K.J., Schmittgen T.D. (2001). Analysis of relative gene expression data using real-time quantitative PCR and the 2(-Delta Delta C(T)) Method. Methods.

